# Acute Effects of Alcohol on the Human Brain: A Resting-State fMRI Study

**DOI:** 10.1155/2015/947529

**Published:** 2015-02-02

**Authors:** Hongyi Zheng, Lingmei Kong, Lanmei Chen, Haidu Zhang, Wenbin Zheng

**Affiliations:** Department of Radiology, The Second Affiliated Hospital, Medical College of Shantou University, Shantou 515041, China

## Abstract

The aim of this study is to assess the value of resting-state fMRI in detecting the acute effects of alcohol on healthy human brains. Thirty-two healthy volunteers were studied by conventional MR imaging and resting-state fMRI prior to and 0.5 hours after initiation of acute alcohol administration. The fMRI data, acquired during the resting state, were correlated with different breath alcohol concentrations (BrAC). We use the posterior cingulate cortex/precuneus as a seed for the default mode network (DMN) analysis. ALFF and ReHo were also used to investigate spontaneous neural activity in the resting state. Conventional MR imaging showed no abnormalities on all subjects. Compared with the prior alcohol administration, the ALFF and ReHo also indicated some specific brain regions which are affected by alcohol, including the superior frontal gyrus, cerebellum, hippocampal gyrus, left basal ganglia, and right internal capsule. Functional connectivity of the DMN was affected by alcohol. This resting-state fMRI indicates that brain regions implicated are affected by alcohol and might provide a neural basis for alcohol's effects on behavioral performance.

## 1. Introduction

Alcohol impairs cognitive function and is associated with a variety of behavioral changes resulting in deficits in perceptual and emotional function. Alcohol consumption has immediate effects on multiple cognitive-motor processing domains and leads to damage of multiple attentional abilities [1]. Previously, functional magnetic resonance imaging (fMRI) has been used to understand the effects of alcohol on the human brain. Five independent critical brain circuits are significantly affected by relatively high levels (blood alcohol concentrations (BAC) = 0.1%) of alcohol, and functional network connectivity between the frontal-temporal-basal ganglia and the cerebellar circuits is specifically disrupted [2, 3]. However, these studies were performed under task-based conditions.

Recently, resting-state fMRI techniques have been applied to demonstrate abnormalities in various neuropsychiatric disorders [4, 5]. The BOLD signal has been confirmed to indirectly reflect neural activity. The default mode network (DMN) has first been observed as a task-negative network, showing increased metabolic demand during the “baseline” activity and has therefore been hypothesized to reflect intrinsic default brain processes [6]. The DMN spans the bilateral posterior cingulate cortex/precuneus (PCC/PCu), retrosplenial cortex (RspC), inferior parietal lobule (IPL), medial prefrontal cortex (mPFC), parts of the hippocampal formation, and the temporal lobe. A research indicated that the PCC/PCu node is particularly noteworthy, since after conditioning it was the only node in the DMN that directly interacted with virtually all other nodes. The PCC/PCu may play a pivotal role in how intrinsic activity is mediated throughout the DMN [7].

Regional homogeneity (ReHo), a novel method that measures the functional connectivity, has been developed to analyze the local synchronization of spontaneous fMRI BOLD signals, reflecting the coherence of spontaneous neuronal activity [8]. Unlike the functional connectivity involved in long-distance interregional temporal correlations of BOLD signals, ReHo, using Kendall's coefficient of concordance (KCC), displays the functional coherence of a given voxel with its nearest neighbors within a single region [8]. The low-frequency (0.01–0.08 Hz) fluctuations (LFFs) of the resting-state fMRI signal were found to be physiologically important, reflecting spontaneous neuronal activity [9]. ALFF was used to study several areas of neuroscience and neurological diseases including healthy aging, schizophrenia, depression, Parkinson's disease, Alzheimer's disease, autism spectrum disorders, and attention deficit hyperactivity disorder [10].

To assess the ability of fMRI to detect the acute effects of alcohol on healthy human brains, we used resting-state fMRI methods to investigate changes in the brain; we hypothesized that acute alcohol administration may alter connectivity measures of the resting-state DMN and have different ReHo and ALFF values in some brain areas when compared with controls.

## 2. Materials and Methods

### 2.1. Subjects

Thirty-two healthy right-handed volunteers (17 men, 15 women; 25–27 years old) were examined by MRI before and after administration of alcohol. To be eligible for the study, potential volunteers were interviewed via telephone and asked a number of questions concerning their general health and medical history, in addition to questions especially related to their history of alcohol use and abuse. All participants provided written informed consent to the study, which was approved by the local ethics committee of the university hospital and institutional review boards. Participants consumed alcohol at a frequency of less than once per week and had no self-reported history of neurological disease, substance abuse, head trauma, CNS tumors, or psychoactive prescriptive medication usage. To ensure that the alcohol dose received in the study would be within the participants' normal range of experience, we excluded very heavy drinkers. To avoid interfering with alcohol absorption, subjects were requested to avoid consuming alcohol for 24 h and refrain from eating for 6 h prior to the study appointment. All participants were given a hand-held breathalyzer test to measure baseline alcohol levels, assuring participants were not already under the influence of alcohol.

#### 2.1.1. Behavioral Evaluation

Before and after alcohol administration, subjects were asked to evaluate their subjective sense of headache, excitement, dizziness, sleepiness, or confusion.

#### 2.1.2. Study Protocol

Subjects passing the screening process were invited to participate in the study. Before alcohol administration, we performed BOLD imaging using MRI to determine the baseline, making each participant serve as a control for the individual alcohol effect. After the examination, each received a dose of 0.65 g of alcohol per kilogram body weight orally within 10 minutes. The alcohol was given in the form of spirit (53° Maotai spirit, 2010, Renhuai, Guizhou, China). All drinks were mixed with some food, such as peanuts. BrAC is an index helping to estimate blood alcohol levels. BrAC was measured before and after each scan session using a hand-held breathalyzer 0.5 hours after alcohol administration. The subjects had to wait for 30 minutes until the BrAC reached its approximate maximum after alcohol administration [11]. Participants were divided into two groups according to the BrAC: a low BrAC group (BrAC = 0–0.36 mg/L) and a high BrAC group (BrAC > 0.36 mg/L). There were 16 participants in each group.

### 2.2. MR Imaging

All anatomical and BOLD-sensitive MRI data were acquired using gradient-echo echo-planar imaging (EPI) sequences in a 1.5T MRI scanner (GE) with an eight-channel-phased array head coil. Foam pads were used to reduce head movements and scanner noise. To measure the individual fMRI data, the imaging parameters were set as follows: slice thickness = 5 mm, slice gap = 1 mm, TR = 2,000 ms, TE = 30 ms, FOV = 24 cm × 24 cm, flip angle = 90°, and matrix = 64 × 64. 180 volumes (20 slices per volume) were acquired during 360 s of an fMRI run. During data acquisition, subjects were required to relax with eyes closed, not to fall asleep, and to move as little as possible. For anatomic data sets, we used a 3D-BRAVO sequence (thickness: 1.4 mm (no gap), TR = 8.2 ms, TE = 1.0 ms, FOV = 24 cm × 24 cm, flip angle = 25°, and matrix = 256 × 256).

### 2.3. Data Processing

Preprocessing of fMRI data was carried out using SPM8 and DPARSF software (http://www.fil.ion.ucl.ac.uk/spm and http://www.restfmri.net/). The first 10 volumes of each functional time series were discarded for the magnetization equilibrium. Head motion parameters were computed by estimating translational and rotational parameters. Each subject had a maximum displacement in a data set that did not exceed ±1.5 mm or ±1.5°. Functional images were normalized to a standard EPI template and interpolated to 3 × 3 × 3 mm cubic voxels. Following this step, all data were copied to two parts: one was smoothed with a 4 mm full width at half maximum (FWHM) for functional connectivity and ALFF analysis and the other one was not smoothed for ReHo analysis. Then, data were temporal band-pass filtered (0.01 < *f* < 0.08 Hz) to reduce the effects of low-frequency drift and physiological high-frequency noise [12], and the linear trend was removed.

### 2.4. DMN Evaluation

The functional connectivity of DMN was calculated using the REST software (http://www.restfmri.net/). The voxel-based correlation approach was used to evaluate the temporally correlated BOLD signal associated with the functional connectivity of the DMN. We make a mask of PCC/PCu which was selected as regions of interest (ROI) from automated anatomical labeling (ALL) atlas. The averaged time course was then computed from each sphere and the correlation analysis was performed in a voxelwise way to generate the functional connectivity of the PCC/PCu. Prior to the correlation analysis, a linear regression was performed to remove the effects of nuisance covariates: the global mean signal, the white matter signal, the cerebrospinal fluid signal, and six head motion parameters. After that, the correlation coefficient maps were converted into *z* maps by Fisher's *r*-to-*z* transform to improve the normality.

### 2.5. ReHo Calculation

ReHo was defined as Kendall's coefficient of concordance (KCC) to study the similarity of the time series within a functional cluster based on the regional homogeneity hypothesis [8]. In the current study, ReHo was used as the KCC of a given voxel with its 26 nearest neighboring voxels. These 27 voxels were defined as a cluster. The individual ReHo map was generated by calculating the KCC in a voxelwise way with free DPARSF software (http://www.restfmri.net/). Then we used a default mask (made from the EPI template in the REST software) to remove nonbrain tissue, and, for standardization purposes, the individual ReHo map was divided by its own mean KCC value within the mask [13, 14].

### 2.6. ALFF Calculation

ALFF was calculated using DPARSF software (http://www.restfmri.net/). For a given voxel, the filtered time series was transformed to a frequency domain with a fast Fourier transform (FFT) to obtain the power spectrum. Then the power spectrum was square-rooted and averaged across 0.01–0.08 Hz at each voxel. This averaged square root was taken as the ALFF [15]. For standardization purposes, the ALFF of each voxel was divided by the global mean ALFF value that was only within the brain and without the background and tissues outside the brain. The standardized ALFF of each voxel should have a value of about 1. This standardization procedure is analogous to that used in PET studies [16].

### 2.7. Statistical Analysis

#### 2.7.1. Functional Connectivity Analysis

To determine brain regions that showed significant positive correlations, one-sample *t*-tests were performed on the individual *z* maps of the PCC/PCu. The statistical threshold was set at *P* < 0.001 and cluster size >6 voxels (AlphaSim corrected). Then, two-sample *t*-tests were used to determine group differences in the functional connectivity with significant correlations within each group. A corrected threshold of *P* < 0.01 and cluster size >18 voxels (AlphaSim corrected) were set to show a significant difference with each group.

#### 2.7.2. ALFF and ReHo Analysis

One-sample two-sided *t*-tests were performed within each group to show whether the standardized KCC value and ALFF differed from the value of one [17]. Then, two-sample *t*-tests were performed to see the ReHo and ALFF difference between the subjects before and after alcohol administration and also between subjects with different BrAC. For ALFF analysis, voxels with a *P* value <0.01 and cluster size >10 voxels were considered to show a significant difference between two groups when analyzed using REST software (http://www.restfmri.net/). For ReHo, a *P* value <0.01 and cluster size >25 voxels were used to indicate a significant difference.

## 3. Results

### 3.1. Effects of Alcohol on the Central and Peripheral Nervous System

Alcohol consumption changed the mood and behavior of the persons tested. Subjects in the low BrAC group complained of headache (*n* = 10), dizziness (*n* = 7), increasing speech (*n* = 9), and feeling tired (*n* = 12). Subjects in the high BrAC group also showed headache (*n* = 14), dizziness (*n* = 13), excitement (*n* = 8), walking unsteadily (*n* = 9), nausea (*n* = 5), and confusion (*n* = 5).

### 3.2. Functional Connectivity Analysis

**Figure 1:**
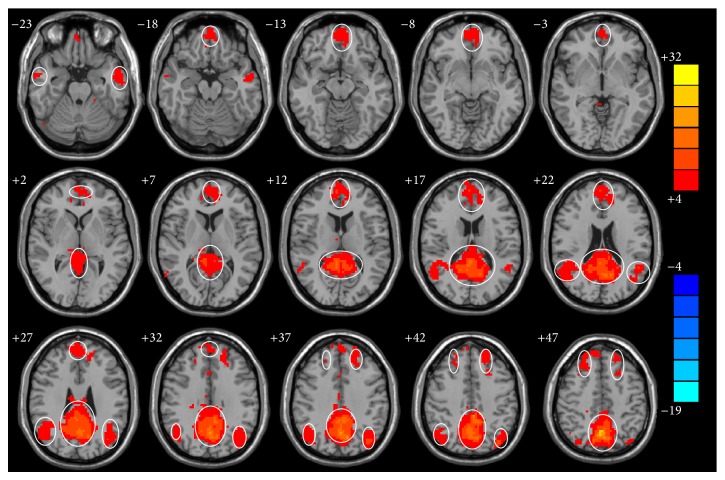
Intragroup maps of connectivity to PCC/PCu of resting-state networks in control group by correlation analysis of resting-state fMRI (*P* < 0.001, AlphaSim, *K*⩾6 voxels). The left side of the images corresponds to the right side of the subjects. T-score bar is shown on the right. Hot colors indicate significant connectivity to PCC/PCu.

The one-sample *t*-test of control group revealed that the intragroup maps of connectivity to PCC/PCu of resting-state network are similar to the DMN. It includes the bilateral posterior cingulate cortex and precuneus, retrosplenial cortex (RspC), medial prefrontal cortex (mPFC), and parts of the temporal lobe which comprise the DMN (Figure 1). The decreasing brain regions of connectivity to PCC/PCu of high and low BrAC group were observed prior to alcohol administration. Using the PCC/PCu as a seed for the functional connectivity analysis, we observed significant decreases of connectivity in the bilateral hippocampal gyrus, left parahippocampal gyrus, left fusiform gyrus, left superior temporal lobe, left superior frontal gyrus, and left rectus gyrus and increases of connectivity in the left cuneus, left occipital lobe, right superior parietal lobe, right superior frontal gyrus, and right cerebellum in high BrAC group as compared with prior to alcohol administration group (Table 1). The low BrAC group also showed decreases of connectivity in the left superior and medial frontal lobe, left occipital lobe, left inferior parietal lobe, and right cerebellum and increases of connectivity in the right cuneus (Table 2). Compared with the low BrAC group, we found that the left rectus gyrus, left cerebellum, right hippocampal gyrus, right superior frontal lobe, bilateral superior temporal lobe, and right fusiform gyrus showed decreases of connectivity and the left medial and superior frontal lobe, right cingulate gyrus, and right cerebellum revealed increases of connectivity in the high BrAC group (Table 3).

### 3.3. ALFF and ReHo Analysis

To investigate the ALFF and ReHo difference, a two-sample *t*-test was performed, which showed a significant difference between the two data sets in certain brain areas. Statistical maps of the two-sample *t*-test were created using a combined threshold of *P* < 0.01 and a minimum cluster size of 10 voxels (Tables 4 and 5) in ALFF and a minimum cluster size of 25 voxels (Tables 7, 8, and 9) in ReHo. A lower threshold of *P* < 0.001 was used to see the difference from different BrAC groups in ALFF (Table 6). Compared with the data obtained prior to alcohol administration, significant positive correlations of the different BrAC groups were observed between ALFF values in the left caudate nucleus, left basal ganglia, left hippocampal gyrus, and left inferior frontal lobe. Negative correlations were also found in the cerebellum, frontal lobe, and temporal lobe. Compared with the low BrAC group, we found a higher ALFF in the right temporal lobe and right parietal lobe and lower ALFF in the bilateral superior frontal lobe (*P* < 0.001, cluster voxels > 10) in the high BrAC group. The higher ReHo values of different BrAC groups in the frontal lobe, cerebellum, right internal capsule, left basal ganglia, left caudate nucleus, left hippocampal gyrus, and left precuneus and lower ReHo values in the frontal lobe, right temporal lobe, right hippocampal gyrus, and left anterior cingulate gyrus (*P* < 0.01, cluster voxels > 25) were found when compared with values obtained prior to alcohol administration. Compared with the low BrAC group, the left midbrain, left middle frontal gyrus, left occipital lobe, right middle temporal lobe, bilateral fusiform gyri, and cerebellum showed a higher ReHo and the bilateral superior frontal lobe, right middle frontal gyrus, right middle and inferior temporal lobe, and cerebellum showed a lower ReHo in the high alcohol group.

## 4. Discussion

Alcohol leads to dysfunction of cognitive control, causing behavioral disinhibition. The mechanism of alcohol action on brain is still not well understood. Recently, a published study used DTI to detect cytotoxic brain edema after acute effects of alcohol on healthy human brain [11]. Other studies using fMRI demonstrated disruption in functional network connectivity with alcohol administration during tasks. These studies using independent component analysis (ICA) identified some circuits including the superior, middle, and orbitofrontal cortex (OFC) and anterior cingulate cortex (ACC), primary/supplementary motor areas, frontal-temporal-basal ganglia, cerebellum, and medial prefrontal, inferior parietal, and lateral temporal cortices while driving under the influence of alcohol [3]. These 5 circuits are significantly affected by relatively high levels (BAC = 0.1%) of alcohol, resulting in both impaired brain function and driving behavior.

This study investigated the effects of alcohol by detecting the functional connectivity of DMN and using ALFF and ReHo. During a resting state, it may be helpful to further understand abnormalities of brain activity in participants under the acute effect of alcohol, because the absence of demanding cognitive activities and instructions makes it more straightforward to compare brain activity across groups that may differ in motivation or cognitive abilities. To determine which brain regions of healthy person are implicated under the influence of acute alcohol, we examined the acute effects of low (<0.36 mg/L BrAC) and high (>0.36 mg/L BrAC) blood alcohol concentrations. We found that many brain regions showed significant differences in functional connectivity, ReHo, and ALFF between the alcohol administration and before alcohol administration; some other brain regions also showed differences between low and high alcohol administration. From these different analyses, we got some different affected brain regions. ReHo and functional connectivity analyses focus on the similarities of intra- and interregional time series, respectively, and ALFF measures the amplitude of regional activity.

**Figure 2:**
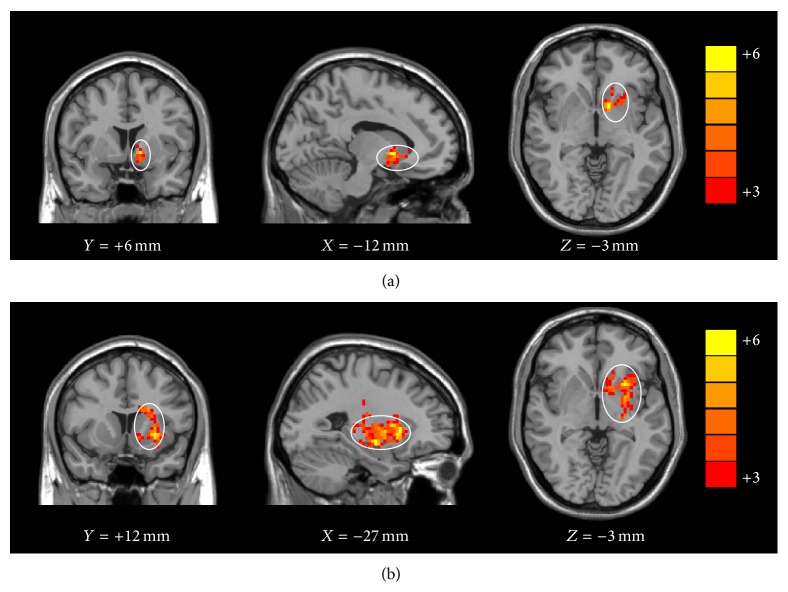
Images showing significant increase in ReHo of left basal ganglia in both low (a) and high (b) BrAC group compared with control group (*P* < 0.01, 25 voxels, uncorrected).

Previous study examining the brain activation during the fMRI Go/No-Go task suggests decrease in brain activation within the basal ganglia and cerebellum, which comprise parts of networks known to be important for movement and cognition [18, 19]. The basal ganglia are associated with a variety of functions, including motor control, behaviors learning, cognitive planning, and emotional functions [20, 21]. The internal capsule is the major route by which the cerebral cortex is connected with the brainstem and spinal cord. The effect on the internal capsule influences the sensory and motor function. The changing ALFF and ReHo in these two regions could affect the control of movement and cognition. Our study showed a higher ReHo of left basal ganglia and right internal capsule in both high and low BrAC group (Figure 2) and a higher ALFF of left basal ganglia in low BrAC group when compared with the control group. But these two regions show no significance when comparing the high BrAC group with the low BrAC group.

Recently, a study indicated significant decrease in connectivity between the frontal-temporal-basal ganglia and cerebellar components during alcohol condition, which might be a vulnerable point to impair one's order cognitive function and motor planning [3]. Functional imaging studies of normal subjects have suggested the activation of the cerebellum as part of neural networks responsible for motor planning, working memory, executive and spatial functions, language, and emotional processes [22, 23]. In this study, the ALFF and ReHo of cerebellum either increased or decreased with alcohol administration. It also showed either increased or decreased functional connectivity in the cerebellum.

There is another region drawing our attention: it is the hippocampal gyrus. The bilateral hippocampal gyri show difference of functional connectivity, ALFF, and ReHo in both high and low BrAC group. A study using DTI to examine the brain indicated that the frontal lobes, thalamus, and middle cerebellar peduncle are especially vulnerable to the effects of alcohol [11]. The hippocampal gyri play a very important content of memory function and are a critical component of medial temporal lobe memory system [23, 24]. Since memory is an important part of individual intelligence, the local connectivity of this region correlates with intelligence. The hippocampal gyrus is commonly linked to target/hazard detection in previous fMRI studies [25].

In the current study, the voxel-based functional connectivity using the PCC/PCu as a seed reveals that the positive correlation brain regions are similar to the DMN. This helps to indicate the PCC/PCu is a central node in the DMN [7]. ALFF and ReHo changes were found in the frontal lobe, parietal lobe, temporal lobe, and hippocampal gyrus, regions that partly comprise the default mode network (DMN). These changes of functional connectivity, ALFF, and ReHo in resting state suggest how alcohol affected the DMN which is responsible for multiple constituent functions, including action, cognition, emotion, interoception, and perception [26, 27]. The decreasing functional connectivity of DMN occurs mainly on the left hemisphere, which may suggest that the left hemisphere of brain is more sensitive to alcohol-related damage than the opposite hemisphere under the resting state. This finding is different from the previous study, which might suggest that the right hemisphere is more vulnerable to alcohol-related damage than the left hemisphere using functional MRI during memory encoding tasks [28]. This finding could also be linked to handedness.

## 5. Conclusion

Resting-state fMRI could detect brain regions including the superior frontal gyrus, cerebellum, hippocampal gyrus, basal ganglia, and internal capsule which were affected by alcohol. These different brain regions which are related to memory, motor control, cognitive ability, and spatial functions might provide a neural basis for alcohol's effects on behavioral performance.

## Figures and Tables

**Table 1:** Brain regions with significant differences of functional connectivity are shown between control and high BrAC group (*P* < 0.01, 18 voxels, AlphaSim corrected).

Brain regions		Voxels	*X* (MNI)	*Y* (MNI)	*Z* (MNI)	*T* value
High BrAC group < control						
Fusiform gyrus	L	61	−36	−9	−45	−5.4005
Parahippocampal gyrus	R	47	−21	3	−33	−4.3482
Hippocampal gyrus	R	20	27	−15	−24	−3.7402
	L	27	−24	−15	−15	−3.735
Superior temporal gyrus	L	68	−51	−6	−27	−4.2768
Rectus gyrus	L	27	3	39	−27	−3.5673
Frontal orbital gyrus	L	25	−15	33	−18	−4.616
Superior frontal gyrus	L	31	−12	48	21	−4.1603
High BrAC group > control						
Cerebellum	R	19	3	−84	−27	4.0301
Cuneus	L	35	−3	−102	−3	4.1482
Occipital gyrus	L	22	−27	−75	12	3.9603
Superior parietal lobe	R	32	15	−51	57	3.7574
Superior frontal gyrus	R	21	6	0	66	3.8591

MNI: Montreal Neurological Institute; L: left; R: right.

*X*, *Y*, and *Z*: coordinates of primary peak locations in the MNI space.

A positive *T* value indicates increased ReHo/ALFF, and a negative *T* value indicates decreased ReHo/ALFF.

**Table 2:** Brain regions with significant differences of functional connectivity are shown between control and low BrAC group (*P* < 0.01, 18 voxels, AlphaSim corrected).

Brain regions		Voxels	*X* (MNI)	*Y* (MNI)	*Z* (MNI)	*T* value
Low BrAC group < control						
Cerebellum	R	56	51	−75	−39	−4.6375
Occipital lobe	L	25	−39	−78	36	−3.6973
Superior frontal gyrus	L	213	−9	24	54	−6.2182
Middle frontal gyrus	L	19	−6	48	−12	−3.3188
Inferior parietal lobe	L	24	−33	−48	54	−3.6218
Low BrAC group > control						
Cuneus	R	31	15	−99	24	3.7814

MNI: Montreal Neurological Institute; L: left; R: right.

*X*, *Y*, and *Z*: coordinates of primary peak locations in the MNI space.

A positive *T* value indicates increased ReHo/ALFF, and a negative *T* value indicates decreased ReHo/ALFF.

**Table 3:** Brain regions with significant differences of functional connectivity are shown between high and low BrAC group (*P* < 0.01, 18 voxels, AlphaSim corrected).

Brain regions		Voxels	*X* (MNI)	*Y* (MNI)	*Z* (MNI)	*T* value
High BrAC < low BrAC group						
Hippocampal gyrus	R	46	24	−18	−27	−3.7115
Rectus gyrus	L	18	0	15	−24	−3.8389
Cerebellum	L	25	−54	−63	−24	−3.7455
Superior temporal gyrus	R	21	45	0	−15	−3.7535
Fusiform gyrus	R	18	42	−30	−21	−4.5647
Superior temporal gyrus	L	44	−45	−6	−15	−3.8467
Superior frontal lobe	R	25	6	48	33	−4.248
High BrAC > low BrAC group						
Cerebellum	R	19	45	−60	−39	3.7278
Cingulate gyrus	R	22	15	6	45	6.7489
Superior frontal lobe	L	31	−9	21	51	5.2161
		40	−27	21	51	5.3112
		18	−15	−9	57	3.5353
Medial frontal lobe	L	35	−27	63	3	4.1768

MNI: Montreal Neurological Institute; L: left; R: right.

*X*, *Y*, and *Z*: coordinates of primary peak locations in the MNI space.

A positive *T* value indicates increased ReHo/ALFF, and a negative *T* value indicates decreased ReHo/ALFF.

**Table 4:** Brain regions with significant differences in ALFF are shown between control and high BrAC group (*P* < 0.01, 10 voxels, uncorrected).

Brain regions		Voxels	*X* (MNI)	*Y* (MNI)	*Z* (MNI)	*T* value
High BrAC group < control						
Superior frontal gyrus	R	654	27	12	63	−6.2074
	L	12	−12	39	51	−3.1304
Inferior frontal gyrus	R	33	54	24	27	−4.113
	R	16	39	33	12	−3.6202
Middle frontal gyrus	R	17	39	30	36	−4.1579
Prefrontal lobe	R	17	48	−6	24	−3.9148
Cerebellum	L	13	−9	−84	−27	−4.0663
Middle temporal gyrus	R	12	63	−39	−9	−3.3521
High BrAC group > control						
Hippocampal gyrus	L	26	−24	0	−15	3.6595
Caudate	L	11	−12	6	24	3.6771

MNI: Montreal Neurological Institute; L: left; R: right.

*X*, *Y*, and *Z*: coordinates of primary peak locations in the MNI space.

A positive *T* value indicates increased ReHo/ALFF, and a negative *T* value indicates decreased ReHo/ALFF.

**Table 5:** Brain regions with significant differences in ALFF are shown between control and low BrAC group (*P* < 0.01, 10 voxels, uncorrected).

Brain regions		Voxels	*X* (MNI)	*Y* (MNI)	*Z* (MNI)	*T* value
Low BrAC group < control						
Cerebellum	L	48	−15	−78	−42	−3.9843
	R	20	6	−48	−51	−3.8325
	R	13	45	−60	−51	−3.5829
	R	13	24	−72	−45	−3.2777
Middle temporal gyrus	R	13	66	−42	6	−3.9775
Low BrAC group > control						
Basal ganglia	L	33	−21	6	−12	4.0375
Inferior frontal gyrus	L	10	−24	24	−21	4.0273

MNI: Montreal Neurological Institute; L: left; R: right.

*X*, *Y*, and *Z*: coordinates of primary peak locations in the MNI space.

A positive *T* value indicates increased ReHo/ALFF, and a negative *T* value indicates decreased ReHo/ALFF.

**Table 6:** Brain regions with ALFF differences are shown between high and low BrAC group (*P* < 0.001, 10 voxels, uncorrected).

Brain regions		Voxels	*X* (MNI)	*Y* (MNI)	*Z* (MNI)	*T* value
High BrAC < low BrAC group						
Superior frontal gyrus	R	68	6	21	60	−5.8642
	R	41	30	30	57	−5.8892
	R	20	27	69	9	−5.7126
	R	10	30	66	−12	−5.8208
	L	32	−12	39	51	−4.9698
	L	24	−21	12	63	−4.6472
High BrAC > low BrAC group						
Middle temporal gyrus	R	15	63	−48	6	5.2313
Parietal lobe	R	11	33	−54	66	6.0077

MNI: Montreal Neurological Institute; L: left; R: right.

*X*, *Y*, and *Z*: coordinates of primary peak locations in the MNI space.

A positive *T* value indicates increased ReHo/ALFF, and a negative *T* value indicates decreased ReHo/ALFF.

**Table 7:** Brain regions with significant ReHo differences are shown between control and high BrAC group (*P* < 0.01, 25 voxels, uncorrected).

Brain regions		Voxels	*X* (MNI)	*Y* (MNI)	*Z* (MNI)	*T* value
High BrAC group < control						
Superior frontal gyrus	R	750	15	21	57	−4.6134
	R	39	12	39	−24	−3.7952
Inferior frontal gyrus	R	37	54	21	18	−4.4388
Hippocampal gyrus	R	39	18	−6	−33	−4.6794
Inferior temporal gyrus	R	30	33	9	−45	−4.2946
High BrAC group > control						
Middle frontal gyrus	L	100	−27	45	3	4.7207
	R	42	12	−21	60	6.4199
Basal ganglia	L	468	−27	12	−3	5.7696
Cerebellum	R	114	9	48	−24	6.8909
Internal capsule	R	39	9	0	6	3.9489

MNI: Montreal Neurological Institute; L: left; R: right.

*X*, *Y*, and *Z*: coordinates of primary peak locations in the MNI space.

A positive *T* value indicates increased ReHo/ALFF, and a negative *T* value indicates decreased ReHo/ALFF.

**Table 8:** Brain regions with a difference in ReHo are shown between control and low BrAC group (*P* < 0.01, 25 voxels, uncorrected).

Brain regions		Voxels	*X* (MNI)	*Y* (MNI)	*Z* (MNI)	*T* value
Low BrAC group < control						
Anterior cingulate	L	33	−6	30	−3	−4.3213
Middle temporal gyrus	R	31	66	−42	6	−4.3893
Hippocampal gyrus	R	30	30	9	−39	−6.1679
Low BrAC group > control						
Basal ganglia	L	89	−12	6	−3	5.9914
Superior frontal gyrus	R	48	3	24	45	3.7264
Internal capsule	R	44	9	0	6	4.6701
Caudate	L	39	−27	6	6	3.9281
Cerebellum	L	25	−36	−42	−42	4.7058
Hippocampal gyrus	L	25	−24	−15	−15	3.9891
Precuneus	L	25	−9	−75	39	3.9728

MNI: Montreal Neurological Institute; L: left; R: right.

*X*, *Y*, and *Z*: coordinates of primary peak locations in the MNI space.

A positive *T* value indicates increased ReHo/ALFF, and a negative *T* value indicates decreased ReHo/ALFF.

**Table 9:** Brain regions with significant differences in ReHo are shown between high and low BrAC group (*P* < 0.01, 25 voxels, uncorrected).

Brain regions		Voxels	*X* (MNI)	*Y* (MNI)	*Z* (MNI)	*T* value
High BrAC < low BrAC group						
Superior frontal gyrus	L	848	−3	21	57	−7.1615
Middle frontal gyrus	R	28	51	51	0	−4.7089
Superior frontal gyrus	R	28	21	15	−15	−3.7462
Inferior temporal gyrus	R	61	45	−9	−36	−5.9454
Middle temporal gyrus	R	40	66	−21	−6	−4.6233
Cerebellum	L	27	−48	−51	−30	−3.9455
High BrAC > low BrAC group						
Middle frontal gyrus	L	25	−30	48	−3	5.0651
Fusiform gyrus	L	105	−30	−57	−3	4.7939
Fusiform gyrus	R	47	24	−78	−15	3.8855
Occipital gyrus	L	37	−27	−87	−9	5.1128
Midbrain		84	−9	−21	−12	5.2828
Middle temporal gyrus	R	65	51	−51	3	4.5885
Cerebellum	R	42	21	−51	−18	4.2606
Cerebellum	L	28	−18	−42	−21	4.6673
Cerebellum	L	35	−27	−66	−24	4.3434

MNI: Montreal Neurological Institute; L: left; R: right.

*X*, *Y*, and *Z*: coordinates of primary peak locations in the MNI space.

A positive *T* value indicates increased ReHo/ALFF, and a negative *T* value indicates decreased ReHo/ALFF.
